# Palmitic Acid Induces Oxidative Stress and Senescence in Human Brainstem Astrocytes, Downregulating Glutamate Reuptake Transporters—Implications for Obesity-Related Sympathoexcitation

**DOI:** 10.3390/nu16172852

**Published:** 2024-08-26

**Authors:** Mahesh Kumar Sivasubramanian, Raisa Monteiro, Manoj Jagadeesh, Priya Balasubramanian, Madhan Subramanian

**Affiliations:** 1 Department of Physiological Sciences, College of Veterinary Medicine, Oklahoma State University, Stillwater, OK 74078, USA; mahesh_kumar.sivasubramanian@okstate.edu (M.K.S.); raisa.monteiro@okstate.edu (R.M.); manoj.jagadeesh@okstate.edu (M.J.); 2 Department of Neurosurgery, The University of Oklahoma Health Sciences Center, Oklahoma City, OK 73104, USA

**Keywords:** glial cells, oxidative stress, senescence, EAAT2, p21/p53, obesity

## Abstract

Obesity has been associated with a chronic increase in sympathetic nerve activity, which can lead to hypertension and other cardiovascular diseases. Preliminary studies from our lab found that oxidative stress and neuroinflammation in the brainstem contribute to sympathetic overactivity in high-fat-diet-induced obese mice. However, with glial cells emerging as significant contributors to various physiological processes, their role in causing these changes in obesity remains unknown. In this study, we wanted to determine the role of palmitic acid, a major form of saturated fatty acid in the high-fat diet, in regulating sympathetic outflow. Human brainstem astrocytes (HBAs) were used as a cell culture model since astrocytes are the most abundant glial cells and are more closely associated with the regulation of neurons and, hence, sympathetic nerve activity. In the current study, we hypothesized that palmitic acid-mediated oxidative stress induces senescence and downregulates glutamate reuptake transporters in HBAs. HBAs were treated with palmitic acid (25 μM for 24 h) in three separate experiments. After the treatment period, the cells were collected for gene expression and protein analysis. Our results showed that palmitic acid treatment led to a significant increase in the mRNA expression of oxidative stress markers (NQO1, SOD2, and CAT), cellular senescence markers (p21 and p53), SASP factors (TNFα, IL-6, MCP-1, and CXCL10), and a downregulation in the expression of glutamate reuptake transporters (EAAT1 and EAAT2) in the HBAs. Protein levels of Gamma H2AX, p16, and p21 were also significantly upregulated in the treatment group compared to the control. Our results showed that palmitic acid increased oxidative stress, DNA damage, cellular senescence, and SASP factors, and downregulated the expression of glutamate reuptake transporters in HBAs. These findings suggest the possibility of excitotoxicity in the neurons of the brainstem, sympathoexcitation, and increased risk for cardiovascular diseases in obesity.

## 1. Introduction

Metabolic syndrome is a conglomeration of conditions that can increase the risk of stroke, heart conditions, and diabetes. Obesity, a serious public health issue, plays an important role in the development of metabolic syndrome. Key conditions associated with metabolic syndrome include increased body fat around the waist, high blood pressure, high cholesterol levels, insulin resistance, dyslipidemia, and high blood sugar, all commonly observed in obesity [1]. The prevalence of obesity almost tripled in the world between 1975 and 2016. According to the World Health Organization (WHO), around 2 billion people are overweight, of which 33% are considered obese. Middle-aged adults in low-income countries and people of all ages and both sexes in high-income countries are affected by obesity [2].

In obesity, excess free fatty acids (FFAs) are released into circulation. Studies have shown that increased FFAs can activate proinflammatory pathways, leading to the increased production of cytokines and chemokines [3,4]. Obesity maintains the body in a state of low-grade chronic inflammation through the production of these proinflammatory molecules. The relationship between obesity and inflammation is a vicious cycle, where more weight gain causes more inflammation and vice versa [5]. Chronic inflammation in obesity can lead to neuroinflammation because of the disruption of the blood–brain barrier, allowing peripheral inflammatory molecules to enter the brain and interact with glial cells, such as astrocytes and microglia. The increased activation and proliferation of these glial cells have been reported in models of obesity [6].

Proinflammatory molecules induce oxidative stress, contributing to neuroinflammation in the brain. Oxidative stress is caused by an increase in reactive oxygen species (ROS) and a decrease in antioxidants, damaging cells at the molecular level and affecting lipids, proteins, and DNA. DNA damage can trigger cellular senescence, an irreversible cell cycle arrest [7,8,9]. Senescent cells produce proinflammatory molecules known as senescence-associated secretory phenotype (SASP) factors, which perpetuate neuroinflammation and create a feedback loop of inflammation and senescence [9,10]. In previous studies from our lab, we found that high-fat-diet (HFD)-induced obese mice produced oxidative stress and cellular senescence in a key brainstem region called the rostral ventrolateral medulla [7,8]. The neurons in this brainstem region are important for the regulation of blood pressure by modulating sympathetic nerve activity (SNA). However, little is known about the contribution of astrocytes in modulating sympathetic overactivity as observed with HFD-induced obesity [7,8].

Astrocytes are the most abundant glial cells and play an important role in neuronal regulation and sympathetic nerve activity. Astrocytes have glutamate reuptake transporters known as Excitatory Amino Acid Transporters (EAATs), which reuptake excess glutamate in the synapse and then convert it into glutamine via glutamine synthetase. This glutamine reenters neurons, is converted back into glutamate, and facilitates excitatory neurotransmission, known as the glutamate-glutamine cycle [11,12]. The downregulation of these transporters could lead to the excess accumulation of glutamate in the synapse and cause excitotoxicity, which in turn could be a contributing factor for sympathoexcitation observed in HFD-induced obesity. Palmitic acid (PA) is a major saturated free fatty acid found in the HFD. Numerous studies have suggested its role in promoting oxidative stress, inflammation, and metabolic dysfunction. Elevated levels of PA are also observed in the plasma of obese individuals. Furthermore, it is well-documented to induce lipotoxic effects in various cell types, including glial cells [3,4,7]. However, whether astrocytes play a role in mediating PA-induced neuroinflammation and the molecular mechanisms that could be involved is undocumented. To answer these questions, we investigated the effect of PA on human brainstem astrocytes.

## 2. Materials and Methods

### 2.1. Cell Culture and Treatments

Human brainstem astrocytes (HBAs) purchased from ScienCell Research Laboratories were used in all the experiments. HBAs were isolated from the human brainstem. These cells were cryopreserved at passage one (P1) and delivered frozen. Each vial had >5 × 10^5^ cells in 1 mL volume. After receiving the cells, they were stored in liquid nitrogen container until they were used. The cells were grown in T-75 flasks with astrocyte media supplied by ScienCell™ and supplemented with Dulbecco’s Phosphate-Buffered Saline (PBS), Fetal Bovine Serum (FBS), Astrocyte Growth Supplement, as well as penicillin and streptomycin. The cultured cells were placed in a 37 °C incubator with 5% CO_2_ until they reached approximately 75–80% confluency. After that, the cells were subcultured in different plates depending on the procedure for which the cells were cultured. Typically, we used passage 3 (P3) for all of our experiments. 

### 2.2. Preparation of BSA-Conjugated Palmitic Acid (PA)

Palmitic acid (500 mM) with ≥99% purity was purchased from Sigma-Aldrich and melted in 1 mL of 100% ethanol at 70 °C. Then, 10% Fraction V fatty acid-free bovine serum albumin (FFA-free BSA) was dissolved in cell culture media, sterile filtered, and incubated at 37 °C. FFA-free BSA obtained from Sigma-Aldrich ass used in the experiment as fatty acids are insoluble in aqueous solutions and can damage the cells. Then, 10 μL of dissolved PA in ethanol was added to 990 μL of 10% FFA-free BSA and vortexed. After vortexing, the conjugate was incubated at 55 °C in a water bath for 15 min. Then, the PA-BSA conjugate was vortexed and kept in the water bath again for 15 min. Then, 10 μL of 100% ethanol with 990 μL of 10% FFA-free BSA was used as a control. The molar ratio of PA-BSA is 3.3:1 [13,14]. 

### 2.3. PA Treatment

After reaching 75–80% confluency, the cells from the T-75 flasks were subcultured in 6-well plates at a seeding density of 15 × 10^4^ for the experiments. Cultured human brainstem astrocytes were treated with palmitic acid at a dose rate of 25 μM for 24 h. The dosage of PA treatment was fixed based on the preliminary experiments from our lab. After the treatment period, the cells were collected in Trizol reagent and processed for real-time PCR analysis.

### 2.4. Oil Red O Staining

Oil Red O staining was performed to stain lipid droplets inside the cells. The Oil Red O stock solution was prepared by dissolving 60 mg of Oil Red O (ORO) in 20 mL of 100% isopropanol and allowed to sit for 20 min. In the meantime, the cell culture media were removed from all the wells and washed twice gently with 1X PBS. After that, the cells were fixed with 10% formalin and incubated for 30 min. The formalin was removed and washed gently with distilled water twice. After that, 60% isopropanol was added to each well and incubated for 5 min. Then the ORO working solution was prepared by adding 3 parts isopropanol to 2 parts of water and added to each well and incubated for 10–20 min. ORO solution is removed after the staining period and washed with distilled water as needed. Then each well was imaged under a light microscope where, in the positive cells, lipid droplets appear red, and nuclei appear blue.

### 2.5. Dihydroethidium Staining 

The dihydroethidium staining was performed in 24 well plates with cells growing on the coverslips. After the treatment period, the culture media were removed, and the plates were rinsed once with 1X PBS. The cells were then incubated with five µM dihydroethidium (DHE; Thermo Fisher Scientific, Waltham, MA, USA) diluted with DMSO and PBS in a light-protected humidified chamber for 30 min at 37 °C. After the incubation period, each well was washed thrice with 1X PBS. Then, the coverslips were inverted onto a slide with prolonged diamond antifade mountant and allowed to rest for 24 h. After that, the slides were imaged under a Biotek Cytation 5 multimode imager for the fluorescent identification of DHE-stained cells.

### 2.6. RNA Extraction and Real-Time PCR Analysis

Human brainstem astrocytes from 6-well plates were collected and lysed in 300 μL Trizol reagent. The Direct-zol RNA Miniprep kit (Zymo Research, Irvine, CA, USA) was used to extract the RNA from the cell culture samples according to the manufacturer’s instructions. Reverse transcription of equal amounts of RNA was performed using the High-Capacity cDNA Reverse Transcription Kit (Applied Biosystems, Waltham, MA, USA) to obtain cDNA. We used 10 ng of cDNA per reaction, and real-time PCR analysis was carried out with iTaq Universal SYBR Green mix (BioRad, Hercules, CA, USA).

The human primers for p16, p21, p53, IL-1α, IL-1β, IL6, IL8, TNF-α, MMP3, MMP13, MCP-1, CXCL1, CXCL10, NQO1, SOD2, CAT, EAAT1, EAAT2, Lamin B1, Plin1, Plin2, and the housekeeping gene GAPDH were designed using Primer3-Blast software and synthesized by Integrated DNA Technologies (primer sequences are listed in Table 1). Real-time PCR data were analyzed using the 2^−ΔΔCT^ method.

### 2.7. Immunofluorescence Staining

Immunofluorescence staining was performed for the DNA damage marker γH2AX and the senescent markers p16 and p21 to determine the presence of senescence in PA-treated cells. For this, we used a 24-well plate with coverslips, and the cells were seeded on top of the coverslips. After the seeding and PA treatment period, the culture media were removed and gently washed with 1X PBS at room temperature (RT). Then, the cells were fixed with 4% paraformaldehyde in PBS at RT for 5 min., after which the coverslips were washed in 1X PBS for 5 min. After that, the permeabilization step was performed with 0.5% Triton X-100 in PBS at RT for 5 min and washed once with 1X PBS for 5 min. Then, the coverslips with cells were blocked with 1% BSA in PBS for 1 h, after which the primary antibody solution (Rabbit polyclonal Gamma H2AX, Catalog No. ab11174, 1:500 dilution; Rabbit polyclonal p16^INK4a^, Catalog No. A0262, 1:100 dilution; and Rabbit polyclonal p21CIP1, Catalog No. A1483, 1:100 dilution) was added to the cells and incubated overnight at 4 °C. The next day, the primary antibody solution was removed, and the coverslips were washed three times in 1X PBS for 5 min each. Then, an appropriate fluorochrome-conjugated secondary antibody (Goat Anti-Rabbit IgG Alexa Fluor 594, Catalog No. ab150080, 1:500 dilution; and Goat Anti-Rabbit IgG Alexa Fluor 488, Catalog No. ab150077, 1:500 dilution) in 1% BSA was added to the cells for 1 h at RT in a dark environment. After that, the cells were gently washed with 1X PBS thrice for 5 min each. After the washing, the coverslips were stained with the nuclear stain DAPI (1:500 dilution in water) for 5 min and washed with 1X PBS twice. Then, the coverslips were mounted on a slide with a drop of prolonged antifade diamond mounting media and allowed to cure overnight. The next day, the slides were imaged using a Biotek Cytation 5 multimodal imager for fluorescent imaging. Finally, the fluorescent intensity of the images was quantified using ImageJ Version 1.54, and the graphs were plotted.

### 2.8. Statistical Analysis

Data were analyzed by Student’s *t*-test and expressed as mean ± SEM. A *p*-value of <0.05 was considered statistically significant. 

## 3. Results

### 3.1. Validation of Palmitic Acid (PA) Treatment in the HBAs

**Figure 1:**
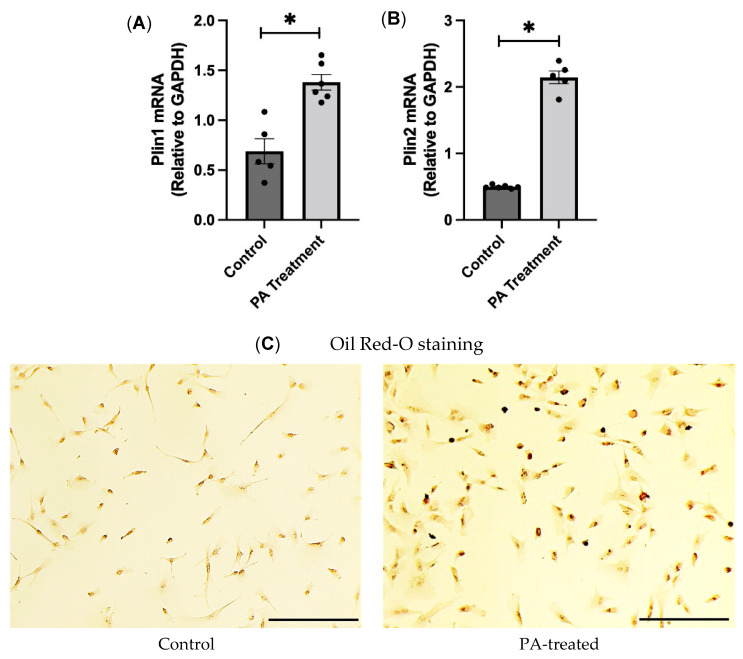
Validation of PA treatment and effects of PA treatment on oxidative stress markers in the HBAs. (**A**,**B**): changes in mRNA levels of lipid droplet associated genes, namely, Plin1 and Plin2 in the human brainstem astrocytes between control and PA-treated group. (**C**): Oil Red-O staining of the control and PA-treated cells at 20X. Scale bar indicates 100 μm. Data are means ± SEM (*n* = 4–6 replicates per group). * significant difference (*p* < 0.05) between the groups.

First, we wanted to test if the palmitic acid treatment was successful in the HBAs. To achieve this, we performed gene expression analysis of the lipid-droplet-associated proteins, perilipin (PLIN) 1 and 2. Perilipins coat the lipid droplets, and increased concentrations of fatty acids will induce lipid droplet accumulation [15]. We found that the mRNA expression of perilipin 1 and 2 was significantly higher in the PA-treated group than in the control groups (Figure 1A, B). Then, we also performed Oil Red O staining to stain the lipid droplets and found that PA-treated cells had a higher number of positive cells, indicating that the palmitic acid treatment was successful and was able to mimic the obese environment in the HBAs (Figure 1C).

### 3.2. PA Induces Oxidative Stress in the HBAs

**Figure 2:**
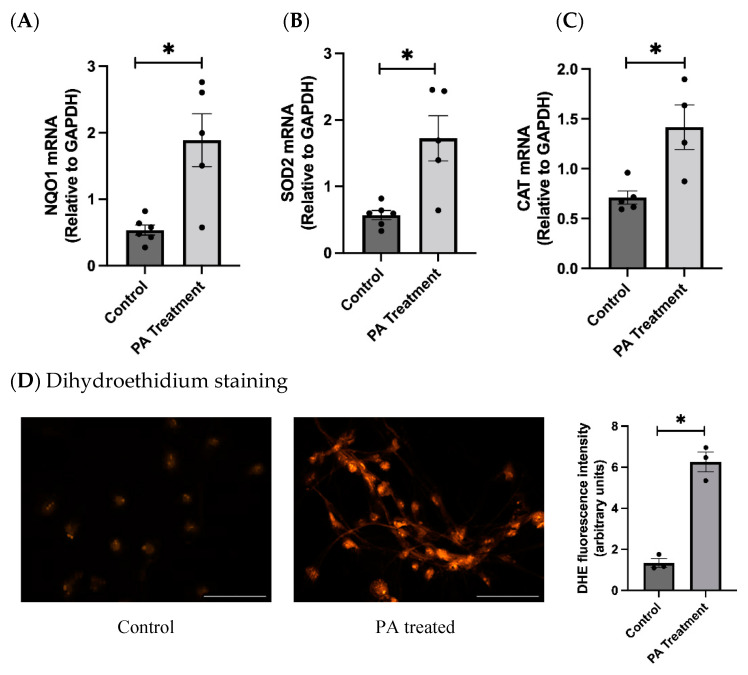
Effects of PA treatment on oxidative stress markers in HBAs. (**A**–**C**): changes in mRNA levels of oxidative stress markers NQO1, SOD2, and CAT in human brainstem between control and PA-treated groups. (**D**): dihydroethidium staining of the control and PA-treated group with bar graphs for fluorescence intensity. Data are means ± SEM (*n* = 3–6 replicates per group). Scale bar indicates 100 μm. * significant difference (*p* < 0.05) between groups.

Studies have shown that PA can enhance the production of reactive oxygen species (ROS) and can cause oxidative stress. PA causes mitochondrial dysfunction and oxidative stress through the NOX pathway. We found that the PA treatment significantly increased the gene expression levels of oxidative stress markers NQO1, SOD2, and catalase (Figure 2A,C). Additionally, we found that the DHE staining of the PA-treated cells indicated that the PA-treated group had significantly higher fluorescence, indicating that PA induces oxidative stress in human brainstem astrocytes (Figure 2D).

### 3.3. PA Induces DNA Damage in the HBAs

**Figure 3:**
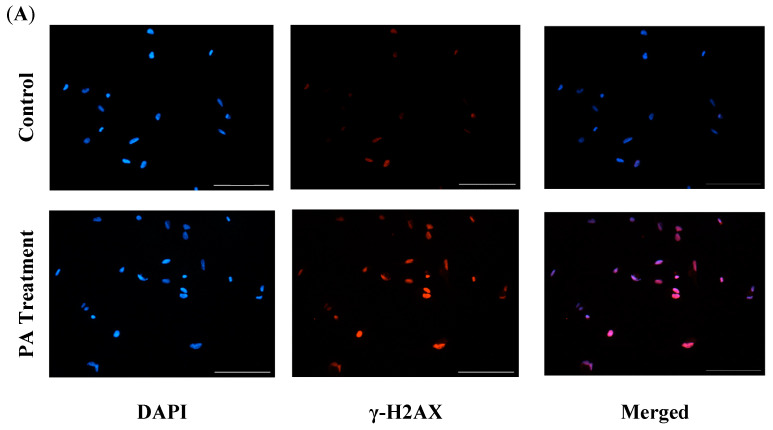

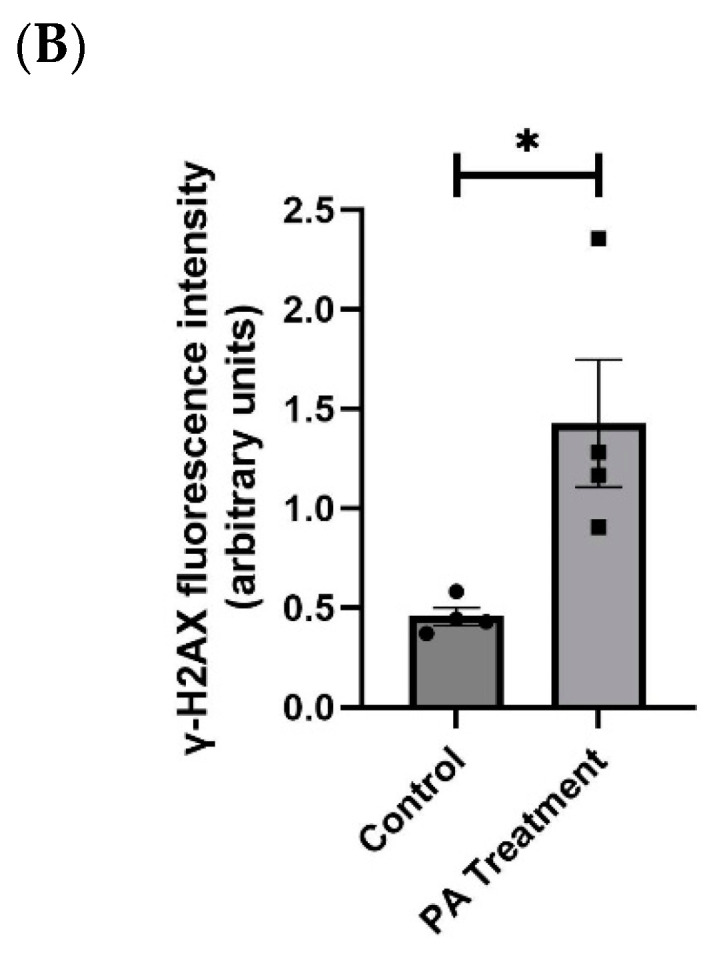
Immunofluorescence staining of Gamma H2AX (γ-H2AX) in the HBAs. (**A**): immunofluorescence staining for the DNA damage marker γ-H2AX in the human brainstem astrocytes between control and PA-treated groups. (**B**): bar graphs showing fluorescence intensity of γ-H2AX between the control and PA-treated groups. Data are means ± SEM (*n* = 4 replicates per group). Scale bar indicates 100 μm. * significant difference (*p* < 0.05) between groups.

After we found that PA treatment induced oxidative stress in the HBAs, we next wanted to see if excess oxidative stress leads to DNA damage. So, we performed immunofluorescence for the DNA damage marker, Gamma H2AX (γ-H2AX), and found that the γ-H2AX fluorescence intensity was significantly upregulated in the PA-treated cells, indicating that the PA treatment induced DNA damage in the HBAs (Figure 3).

### 3.4. PA Causes Cellular Senescence and SASP in the HBAs

**Figure 4:**
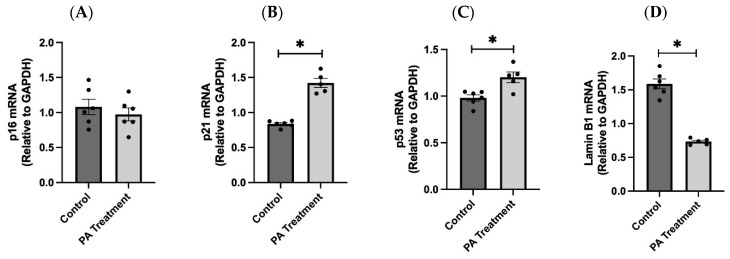
PA-induced oxidative stress causes cellular senescence in the HBAs. (**A**–**D**): Changes in the mRNA levels of senescence markers p16, p21, p53 and the nuclear envelope gene Lamin B1 in the human brainstem astrocytes between control and PA-treated groups. Data are means ± SEM (*n* = 5–6 replicates per group). * Significant difference (*p* < 0.05) between the groups.

PA treatment has been shown to induce cellular senescence, a state of irreversible cell cycle arrest, through oxidative stress and the resulting DNA damage. Consistent with this earlier finding, we observed that PA treatment in the HBAs significantly increased the gene expression levels of senescence markers p21 and p53 (Figure 4A,C), along with elevated expression of oxidative stress and DNA damage markers. However, we did not observe a significant difference in p16 mRNA expression between the groups. In addition, Lamin B1, a component of the nuclear envelope, was significantly downregulated in the PA treatment group consistent with the senescence (Figure 4D). 

**Figure 5:**
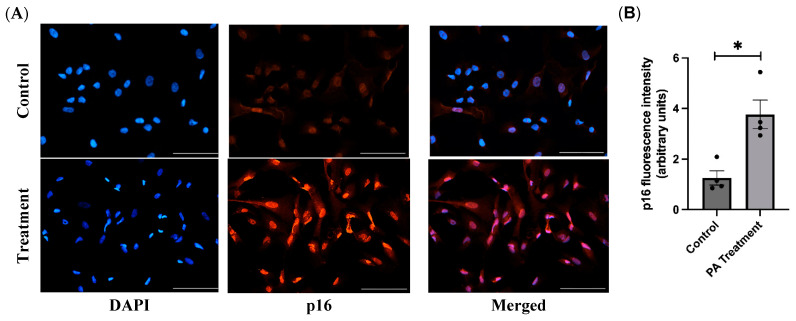
Immunofluorescence staining of p16^INK4a^ in the HBAs. (**A**): immunofluorescence staining for the senescence marker p16^INK4a^ in the human brainstem astrocytes between control and PA-treated groups. (**B**): bar graphs showing fluorescence intensity of p16^INK4a^ between the control and PA-treated groups. Data are means ± SEM (*n* = 4 replicates per group). Scale bar indicates 100 μm. * significant difference (*p* < 0.05) between the groups.

**Figure 6:**
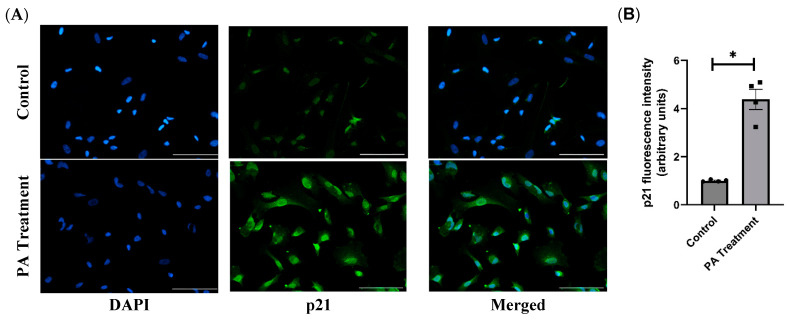
Immunofluorescence staining of p21CIP1 in the HBAs. (**A**): immunofluorescence staining for the senescence marker p21CIP1 in the human brainstem astrocytes between control and PA-treated groups. (**B**): bar graphs showing fluorescence intensity of p21CIP1 between the control and PA-treated groups. Data are means ± SEM (*n* = 4 replicates per group). Scale bar indicates 100 μm. * significant difference (*p* < 0.05) between the groups.

By immunofluorescence staining of the PA-treated cells with p16 and p21 antibodies, we found that the PA-treated cells have significantly higher p16 and p21 levels, further confirming that PA treatment causes cellular senescence (Figure 5 and Figure 6).

**Figure 7:**
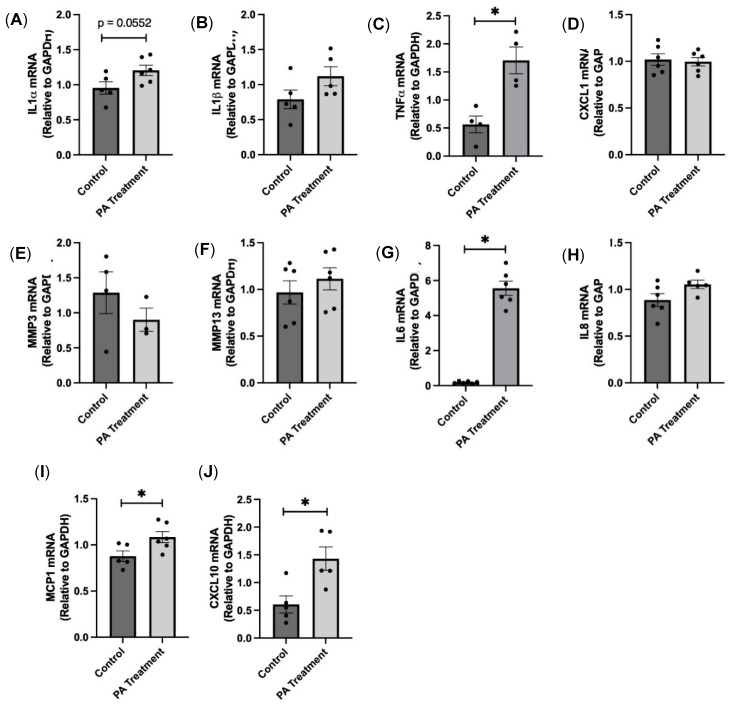
PA-induced senescence causes SASP factor production in the HBAs. (**A**–**J**): changes in the mRNA levels of various proinflammatory SASP factors in the human brainstem astrocytes between control and PA-treated groups. Data are means ± SEM (*n* = 5–6 replicates per group). * significant difference (*p* < 0.05) between the groups.

Senescent cells, in addition to arresting growth, also secrete various proinflammatory factors, known as SASP factors, which can affect neighboring cells in a paracrine fashion. We found that there is a significant increase in the gene expression levels of SASP factors IL1α, TNFα, IL6, MCP1, and CXCL10 (Figure 7), indicating that SASP factors, which are a potential source of neuroinflammation, are produced with PA treatment.

### 3.5. PA Downregulates Glutamate Reuptake Transporter Expression in the HBAs

**Figure 8:**
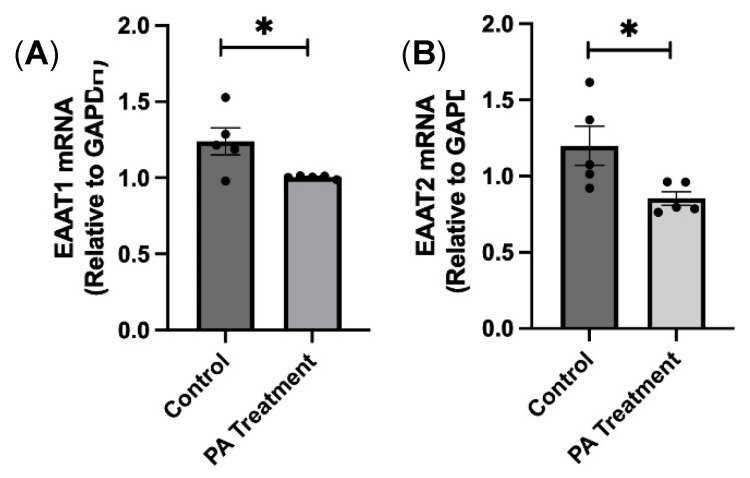
PA treatment downregulates glutamate reuptake transporter expression in the HBAs. (**A**,**B**): changes in the mRNA levels glutamate re-uptake transporters, EAAT1 and EAAT2 in the human brainstem astrocytes between control and PA-treated groups. Data are means ± SEM (*n* = 4–6 replicates per group). * significant difference (*p* < 0.05) between the groups.

The downregulation of glutamate reuptake transporters can cause excitotoxicity due to excess glutamate in the synapse, which in turn can increase sympathetic nerve activity to the end organs and damage them. We wanted to test if PA treatment downregulates these glutamate transporters. We found that the mRNA expression levels of EAAT 1 and EAAT 2 were significantly downregulated in the PA-treated group compared with the controls, indicating that PA downregulates glutamate reuptake transporter expression (Figure 8).

## 4. Discussion

The major findings of this study include: (i) palmitic acid treatment increased the expression of oxidative stress and DNA damage markers in human brainstem astrocytes; (ii) palmitic acid elevated the mRNA and protein expression of cellular senescence markers; (iii) palmitic acid upregulated the mRNA expression of SASP factors; and (iv) palmitic acid treatment induced the downregulation of glutamate reuptake transporters, EAAT1 and EAAT2.

It is widely known that elevated free fatty acids, such as palmitic acid (PA), in circulation can contribute to increased oxidative stress in an obese environment [3,4]. Our findings align with these studies, demonstrating that PA treatment significantly increased oxidative stress markers such as NQO1, SOD2, and CAT. Similar to the work by Ogrodnik et al. (2019), which showed that obesity leads to DNA damage and accumulation of senescent glial cells, we found that PA-induced oxidative stress in the HBAs also resulted in DNA damage, as determined by γH2AX staining. Our study further extends these findings by showing that DNA damage occurring in the HBAs due to oxidative stress can stimulate a DNA damage response (DDR), which, when sustained, can cause them to enter a state of cellular senescence [16,17].

Cellular senescence is a complex biological process characterized by irreversible growth arrest, involving cell cycle inhibitors (p16, p21, and p53) and a proinflammatory state (SASP factors) [18]. With PA treatment, we found that senescence markers p21 and p53 were increased in the HBAs. We further explored the cell cycle inhibitors at the protein level and confirmed that both p16 and p21 were significantly upregulated with PA treatment, similar to other obese animal studies [19]. To examine whether senescent glial cells created a proinflammatory state [20,21], we measured the levels of SASP factors such as TNFα, IL6, MCP1, and CXCL10 and found them to be upregulated after PA treatment. Other studies have shown that chronic exposure to SASP factors could cause damage to healthy cells in a paracrine manner and could be responsible for tissue dysfunction in obesity and aging [22].

One significant finding from our current study is the observation that PA treatment leads to the downregulation of glutamate reuptake transporters (EAAT1 and EAAT2). The importance of glutamate reuptake transporters has been described in various studies, and the dysregulation of glutamate homeostasis is a key pathological feature, even in the development of neurodegenerative diseases [23,24]. The astrocytes closely associated with the synapse are responsible for the synthesis of glutamine, which eventually produces glutamate. Under normal circumstances, excess glutamate is taken up by astrocytes using the reuptake transporters [25]. However, the reduced reuptake transporters after PA treatment suggests that there would be an excess accumulation of glutamate at the synapse, which could be responsible for chronic sympathoexcitation as seen in high-fat diet-induced obesity [7,26,27]. To summarize, this study provides evidence that palmitic acid treatment increased the oxidative stress, cellular senescence, and downregulation of glutamate reuptake transporters in HBAs.

One of the main limitations of this present study is that the effects observed are only in the HBAs in isolation, without exploring interactions with neurons and microglia. To overcome this limitation, our future studies will employ co-culture and/or animal experiments to investigate glia–neuron crosstalk and glia–glia interaction. The second limitation is that we only used palmitic acid in our experiments and didn’t compare it with any other fatty acids, such as oleic acid, or other stressors like glucose, insulin, and H2O2. These will be explored in our future studies. 

## 5. Conclusions

Our findings demonstrate that elevated PA in obesity can impair astrocyte function, leading to oxidative stress, DNA damage, cellular senescence, and downregulation of glutamate reuptake transporters. These changes can potentially disrupt glutamate homeostasis, contributing to excitotoxicity and neurodegeneration. Therefore, modulating these transporters could be a novel therapeutic approach to reduce obesity-related sympathetic overactivity and its associated cardiovascular complications. However, future studies in animals would be required to reaffirm these preliminary in vitro findings. 

## Figures and Tables

**Table 1:** Primer genes and their gene accession numbers for real-time PCR analysis.

Human Gene	Forward Primer	Reverse Primer	Gene Accession No.
*p16*	GATCCAGGTGGGTAGAGGGTC	CCCCTGCAAACTTCGTCCT	NM_058197.5
*p21*	TGTCCGTCAGAACCCATGC	AAAGTCGAAGTTCCATCGCTC	NM_001374511.1
*p53*	CAGCACATGACGGAGGTTGT	TCATCCAAATACTCCACACGC	NM_001407269.1
*IL1α*	CGCCAATGACTCAGAGGAAGA	AGGGCGTCATTCAGGATGAA	NM_000575.5
*IL1β*	AATCTGTACCTGTCCTGCGTGTT	TGGGTAATTTTTGGGATCTACACTCT	NM_000576.3
*IL6*	CCGGGAACGAAAGAGAAGCT	GCGCTTGTGGAGAAGGAGTT	NM_000600.5
*IL8*	CTTGGCAGCCTTCCTGATTT	TTCTTTAGCACTCCTTGGCAAAA	NM_001354840.3
*TNF-α*	CCCAGGGACCTCTCTCTAATCA	AGCTGCCCCTCAGCTTGAG	NM_000594.4
*MMP3*	GACACCAGCATGAACCTTGTT	GGAACCGAGTCAGGTCTGTG	NM_002422.5
*MMP13*	GGCTCCGAGAAATGCAGTCTTTCTT	ATCAAATGGGTAGAAGTCGCCATGC	NM_002427.4
*MCP-1*	AATCAATGCCCCAGTCACCT	CTTCTTTGGGACACTTGCTGC	NM_002982.4
*CXCL1*	GAAAGCTTGCCTCAATCCTG	CACCAGTGAGCTTCCTCCTC	NM_001511.4
*CXCL10*	AACCTCCAGTCTCAGCACCATGAA	AGGTACAGCGTAAGGTTCTAGAGAG	NM_001565.4
*NQO1*	ACTGATCGTACTGGCTCACTC	AGTTCATGGCATAGAGGTCCG	NM_001025434.2
*SOD2*	GTTGGGGTTGGCTTGGTTTC	GTTCCTTGCAGTGGATCCTGA	NM_001322819.2
*CAT*	CTTTCTGTTGAAGATGCGGCG	AGTCCAGGAGGGGTACTTTCC	NM_001752.4
*EAAT1*	TGCCCTGGGTCTAGTTGTCT	CCAGTCTCATGATGGCTTCGT	NM_004172.5
*EAAT2*	CTTGGCATCTCCCATCCACC	GCTGGAGATGATTAGAGGGAGAA	NM_001252652.2
*Lamin B1*	AAGGCGAAGAAGAGAGGTTGAAG	GCGGAATGAGAGATGCTAACACT	NM_001198557.2
*Plin1*	CAGAAACAGCATCAGCGTTCC	CAGCAAATTCCGCAGTGTCTC	NM_001145311.2
*Plin2*	TCACAGGGGTGATGGACAAG	TTTCTACGCCACTGCTCACG	NM_001122.4
*GAPDH*	GCCGTCTAGAAAAACCTGCC	ACCACCTGGTGCTCAGTGTA	NM_001357943.2

## Data Availability

Data are contained within the article.
